# Optimization of bottom structure layout and key parameters in block caving: a case study of the Cukaru Peki Copper-Gold Mine in Serbia

**DOI:** 10.1038/s41598-026-56171-8

**Published:** 2026-06-06

**Authors:** Zhili Su, Min Huang, Yongding Su, Weijun Qiang, Zhe Chen, Chenhong Wang, Pengyu Guo, Zongze Li

**Affiliations:** 1 Zijin Mining Group Co., Ltd, Shanghang, 364200 Fujian China; 2 Zijin (Changsha) Engineering Technology Co., Ltd, Changsha, 410036 Hunan China; 3 State Key Laboratory of Coal Mine Disaster Dynamics and Control, School of Resources and Safety Engineering, Chongqing University, Chongqing, 400044 China; 4 Division of Mining and Geotechnical Engineering, Luleå University of Technology, Luleå, 97187 Sweden

**Keywords:** Block caving method, Bottom structure, Undercut distance, Parameter optimization, Numerical simulation, Energy science and technology, Engineering, Solid Earth sciences

## Abstract

The stability of the bottom structure and the scientific management of ore drawing are critical to the successful application of the block caving method. In view of the engineering conditions of the Lower Orebody of the Cukaru Peki Copper-Gold Mine operated by Serbia Zijin Copper Doo Bor characterized by great burial depth, high in-situ stress, and large-scale mechanized ore extraction. This study systematically optimizes the layout form and key geometric parameters of the bottom structure through a comprehensive approach combining engineering analogy, theoretical analysis, numerical simulation, and orthogonal experimental design. The results indicate that, in terms of the layout at the extraction level, the branching herringbone configuration is the optimal scheme. It not only ensures sufficient overlap of draw zones and stability of the sill pillar, but also better accommodates the efficient operation of electric load–haul–dump (LHD) machines. Regarding key parameter determination, coupled analyses of vertical and planar geometric relationships demonstrate that the optimal vertical distance between the extraction level and the undercut level is 20 m. The spacing of drawpoint crosscuts and drifts is determined to be 32 m and 18 m, respectively. The optimized dimensions of the ore pass are identified as 5.0 m for the lower opening width and 14 m for the upper opening width, which significantly reduce the incidence of drawpoint hang-ups. For the undercutting scheme, an advancing undercut method combined with an inclined narrow-section layout is recommended, with an undercut height of 6 m. Furthermore, numerical simulations reveal the influence of the advanced undercut distance on stress redistribution. It is recommended that a reasonable distance of 30–36 m be maintained between ore pass operations and the advancing undercut front to effectively mitigate structural damage caused by high stress concentrations in the bottom structure. The parameter system established in this study provides a scientific basis for the safe and efficient exploitation of the Cukaru Peki deposit operated by Serbia Zijin Copper Doo Bor and offers important engineering guidance for bottom structure design in similar deep block caving mines.

## Introduction

With the gradual depletion of shallow mineral resources, deep mining has become an inevitable trend in the global mining industry^1,2^. Owing to its significant advantages of large-scale production, high efficiency, and low cost, the block caving method has become the preferred technique for exploiting deep, low-grade, and super-thick ore bodies^3–5^. The core principle of this method is to utilize the in-situ stress and gravitational forces of the rock mass to induce continuous caving of the ore from bottom to top, thereby achieving gravity-driven ore flow^6^. However, as mining depths extend to the kilometer scale, the bottom structure—regarded as the “heart” of block caving—is facing unprecedented challenges. Extremely high ground stress (25–30 MPa), elevated rock temperatures (> 45 °C), and complex tectonic stress environments can easily result in severe plastic deformation and roof collapse within the bottom structure^7–9^. The layout form and geometric parameters of the bottom structure are therefore critical factors determining production safety and extraction efficiency.

Considerable research has been conducted on bottom structure layout schemes. The Teniente layout, developed at the El Teniente Mine in Chile, has demonstrated strong robustness in ground pressure management. However, Xiao et al. pointed out that its compact roadway arrangement restricts the operational efficiency of modern large-scale LHD machines^10^. In contrast, the branching herringbone layout applied at the Northparkes Mine in Australia achieves a better balance between mechanical stability and equipment maneuverability^11^. Domestic scholars such as Zhong et al. and An et al. have suggested that branching herringbone structures exhibit superior compressive resistance in deep mining conditions^12,13^. Nevertheless, most existing studies on structural configurations are based on shallow to medium-depth conditions or conventional in-situ stress environments^14–16^. Their design concepts largely rely on empirical analogy or single-factor analysis methods, lacking a systematic understanding of structural response mechanisms under ultra-deep (> 1200 m) high-stress conditions. In particular, under large-span conditions (pillar spacing > 30 m) and near-vertical orebody settings, the failure mechanisms of the bottom structure induced by the coupling effects of excavation unloading and tectonic stress have not yet been fully clarified. This, to some extent, limits the engineering applicability of existing design methodologies.

Regarding the optimization of key geometric parameters, both domestic and international researchers have conducted studies based on ore flow theory and numerical simulation. For vertical parameters, foreign mines often adopt empirical criteria proposed by Mathieu, setting the sill pillar height (H₁) at 6–8 m^17^. Chen et al. attempted to establish a dynamic design approach linking sill pillar height to stress concentration characteristics induced by undercutting^18^. However, for ultra-deep ore bodies, quantitative evaluation of sill pillar safety margins remains inadequate. In terms of planar parameters, the spacing of drawpoint crosscuts is primarily determined based on granular flow theory. Henning and Mitri demonstrated that crosscut spacing significantly affects ore dilution^19^. Although domestic studies have performed simulations using FLAC 3D and PFC, most focus on single-variable analyses and lack investigation of the coupled effects among burial depth, ground stress, and structural dimensions^20,21^. Furthermore, while evidence suggests that the advancing undercut method is superior to pre-undercutting in maintaining bottom structure stability refined simulation and evolutionary analysis of the optimal advanced undercut distance under ultra-deep and steeply dipping ore body conditions are still at an early stage^22–24^.

Although extensive studies have been conducted by domestic and international researchers on bottom structure configurations and geometric parameter optimization, several key issues remain unresolved. First, there is a lack of a systematic design theory for bottom structures under ultra-deep and high-stress conditions. Existing approaches largely rely on empirical analogy and are difficult to adapt to the complex in-situ stress environments at burial depths exceeding 1000 m. Second, the coupling mechanisms among structural configuration, geometric parameters, and stress evolution remain unclear. In particular, under the combined effects of excavation-induced unloading and tectonic stress, the response behavior and instability mechanisms of bottom structures have not yet been sufficiently revealed. Third, optimization of drawpoint and undercutting parameters lacks a unified mechanical analysis framework. Most existing studies are based on single-factor analyses or empirical criteria, making it difficult to achieve coordinated optimization between structural stability and ore recovery efficiency. Therefore, it is imperative to develop a bottom structure optimization methodology that comprehensively accounts for high in-situ stress conditions, structural layout configurations, and mining-induced disturbances. Such an approach should elucidate the intrinsic relationship between structural stability and ore flow behavior, and ultimately enable the coordinated improvement of both structural safety and extraction efficiency.

**Fig. 1:**
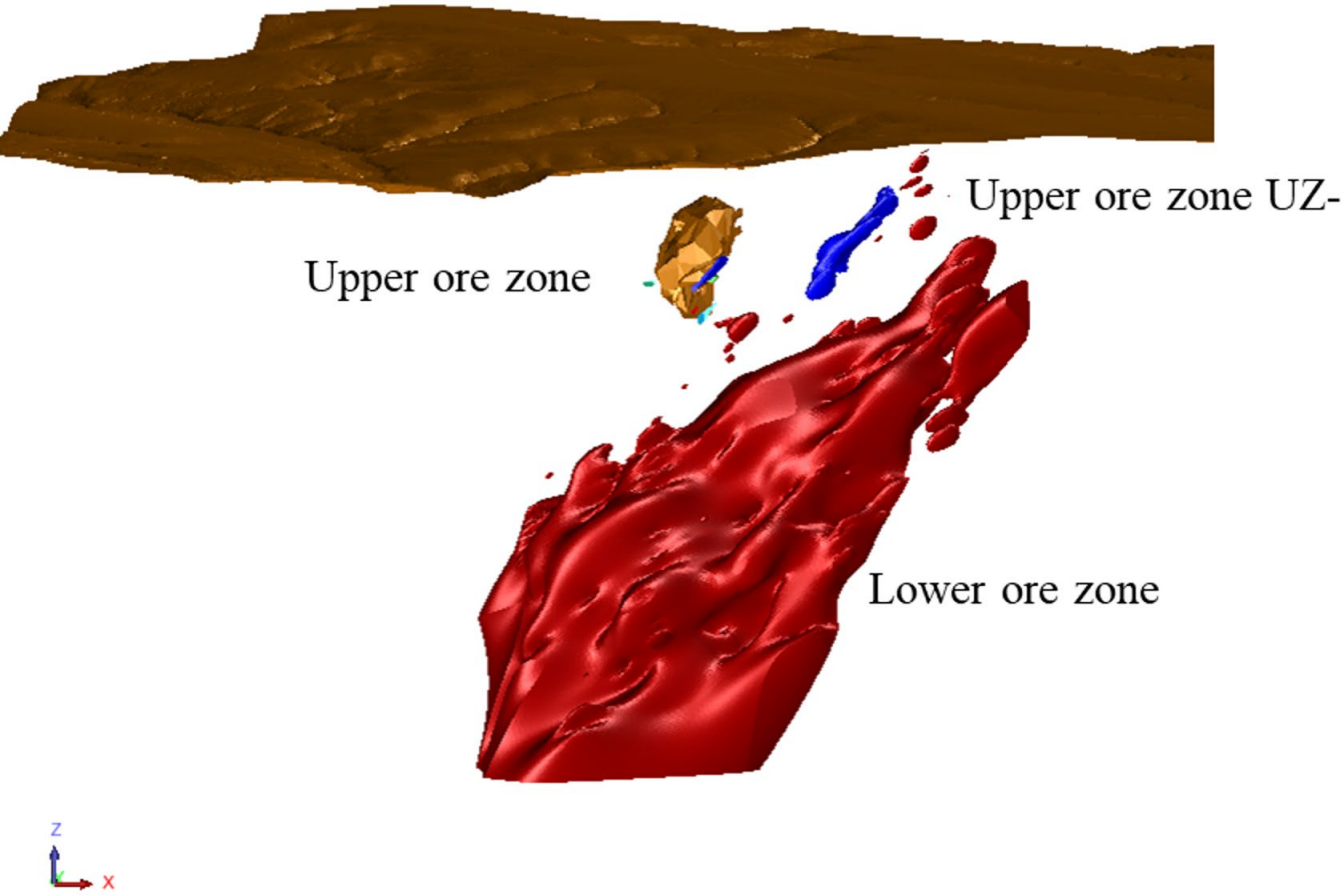
Relative positions of the upper and lower ore zones.

The Cukaru Peki Copper-Gold Mine of Serbia Zijin Copper Doo Bor is located approximately 6 km south of the city of Bor in eastern Serbia and falls under the administrative jurisdiction of Bor. The deposit is a super-large copper–gold deposit within the Mediterranean porphyry metallogenic belt. It is divided into the Upper Orebody and the Lower Orebody, of which the Lower Orebody is a porphyry-type copper–gold deposit characterized by large scale and abundant resources. Its relative location is shown in Fig. 1.

The Lower Orebody is mined at depths ranging from 1200 to 2200 m, representing a typical ultra-deep, high-stress deposit. The integrity control of the sill pillar has become a critical bottleneck restricting mine development. Against this background, this study takes the Lower Orebody as the engineering case and adopts a combined approach of engineering analogy, theoretical calculation, and numerical simulation. First, suitable bottom structure layouts under these specific conditions are comparatively evaluated and optimized. Subsequently, following the logical framework of “vertical parameters–planar parameters–undercutting scheme,” key parameters-including the vertical distance between the extraction and undercut levels, drawpoint crosscut spacing, ore pass geometry, sill pillar height, and advanced undercut distance-are systematically analyzed and determined. The objective of this research is to establish an optimized bottom structure design system applicable to ultra-deep, steeply dipping ore bodies, thereby providing scientific guidance and technical support for safe and efficient extraction in similar deep mining operations.

The rock mass was divided into different depth intervals, and the average values of the physical and mechanical parameters were statistically analyzed and summarized in Table 1. Among them, the depth range of 200–420 m corresponds to the rock mass section without orebody occurrence; the depth range of 420–860 m corresponds to the rock mass section containing the main orebody of the upper ore zone; the depth range of 860–1300 m corresponds to the rock mass section containing the orebody of Phase I of the sublevel caving project in the lower ore zone; and the depth range of 1300–1800 m corresponds to the rock mass section containing the orebody of Phase II of the sublevel caving project in the lower ore zone.

**Table 1:** Physical and mechanical parameters of the rock mass at different depth intervals.

Depth –m	Sample	Bulk density γ–kN m ^3^	Compressive strength σ_c_–MPa	Tensile strength σ_t_–MPa	Cohesion c–MPa	Friction angle φ–°	Elastic modulus E_m_–MPa	Poisson’s ratioµ
200–300	7	26.17	39.77	5.78	5.16	42.60	11164.7	0.25
300–400	12	26.44	41.25	6.75	8.27	46.71	16675.4	0.29
400–500	23	27.04	44.68	7.67	9.19	44.63	17544.4	0.28
500–600	12	27.40	52.66	7.60	9.97	46.67	20227.6	0.25
600–700	14	26.81	47.26	6.47	8.73	46.86	17614.1	0.25
700–800	14	27.25	68.84	9.77	12.93	46.57	25211.3	0.23
800–900	15	27.33	75.84	9.41	13.34	50.27	26696.4	0.24
900–1000	20	27.54	80.36	9.53	13.23	50.16	27904.6	0.26
1000–1100	38	27.47	64.40	8.63	11.22	47.56	22933.5	0.29
1100–1200	27	27.21	58.02	8.69	11.14	46.04	21413.4	0.26
1200–1300	15	28.14	72.54	8.66	12.29	48.91	24058.3	0.28
1300–1400	18	28.63	50.11	7.30	8.81	47.90	17354.7	0.28
1400–1500	13	28.45	37.97	7.52	8.42	42.30	15184.6	0.29
1500–1600	4	28.95	30.91	6.94	7.32	41.00	13264.0	0.30
1600–1700	4	28.04	30.21	5.52	6.46	42.67	13003.5	0.31
1700–1800	1	27.80	39.78	7.24	8.50	44.00	16625.0	0.27
200–420	25	26.52	42.08	6.80	7.64	44.88	15831.6	0.27
420–860	88	27.03	54.81	7.75	9.70	45.92	19349.8	0.26
860–1300	84	27.57	67.81	8.89	11.95	48.17	24012.2	0.27
1300–1800	40	28.53	41.93	7.22	8.38	44.21	15954.5	0.29
200–1300	197	27.26	62.03	8.44	11.05	47.19	22158.1	0.27
200–1800	237	27.48	58.54	8.27	10.67	46.76	21227.9	0.27

According to the field engineering geological investigation and the detailed geological exploration report of the mining area, the structural planes developed in the mine are mainly tectonic structural planes, followed by primary structural planes. Among the tectonic structural planes, joints, fractures, and faults are dominant, with joints and fractures widely distributed in different rock groups. Based on the development scale and characteristics of the major structural planes (joints and fractures), and according to the structural plane classification method, the major structural planes were identified as Grade II and Grade IV structural planes. Joints and fractures are well developed, most of which are closed, while some fractures are open with apertures ranging from 0.1 to 2 mm and filled with quartz. The joint surfaces are rough and slightly weathered. This rock stratum is a non-aquifer, and groundwater mainly exists as seepage through structural fractures. The joint and fracture surfaces are generally dry or moist. The joint spacing of 0–0.2 m accounts for the highest proportion, reaching 50.3%, followed by the 0.2–0.4 m interval. Together, these two intervals account for 81.4%, indicating that joints are highly developed in this rock group. The dominant joint sets in this rock group are 207°∠36°, 8°∠23°, and 152°∠44°.In this study, the MRMR-2000 evaluation system was adopted, and the rock mass quality evaluation results for different burial depth ranges are summarized in Table 2.

**Table 2:** Rock mass quality evaluation results for different depth intervals.

Lithology	Depth–m	RQD average	RQD rock quality classify-cation	RMR average	RMR rock quality classify-cation	MRMR average	MRMR rock quality classify-cation	Comprehensive evaluation results of rock quality
200–300	77.16	II	38.6	IV	–	–	IV	200–300
300–400	74.43	III	37	IV	26.1	IV	IV	300–400
400–500	67.27	III	37.6	IV	29.1	IV	IV	400–500
500–600	69.13	III	43.6	III	32.9	IV	IV	500–600
600–700	67.37	III	40	IV	33.3	IV	IV	600–700
700–800	72.71	III	40	IV	31.9	IV	IV	700–800
800–900	76.39	II	48.1	III	38.7	IV	IV	800–900
900–1000	79.36	II	52.1	III	41.9	III	III	900–1000
1000–1100	77.44	II	46.9	III	34.8	IV	IV	1000–1100
1100–1200	76.13	II	43	III	40.7	IV	IV	1100–1200
1200–1300	79.84	II	56	III	47.2	III	III	1200–1300
1300–1400	87.59	II	49.5	III	41.4	III	III	1300–1400
1400–1500	82.22	II	55.0	III	–	–	III	1400–1500
1500–1600	80.21	II	54.1	III	–	–	III	1500–1600
1600–1700	68.44	III	52.8	III	–	–	III	1600–1700
1700–1800	59.77	III	51.9	III	–	–	III	1700–1800
1800–1900	59.77	III	51.4	III	–	–	III	1800–1900
1900–2000	69.13	III	51.2	III	–	–	III	1900–2000
2000–2100	87.34	II	50.9	III	–	–	III	2000–2100

## Selection of bottom structure layout

**Fig. 2:**
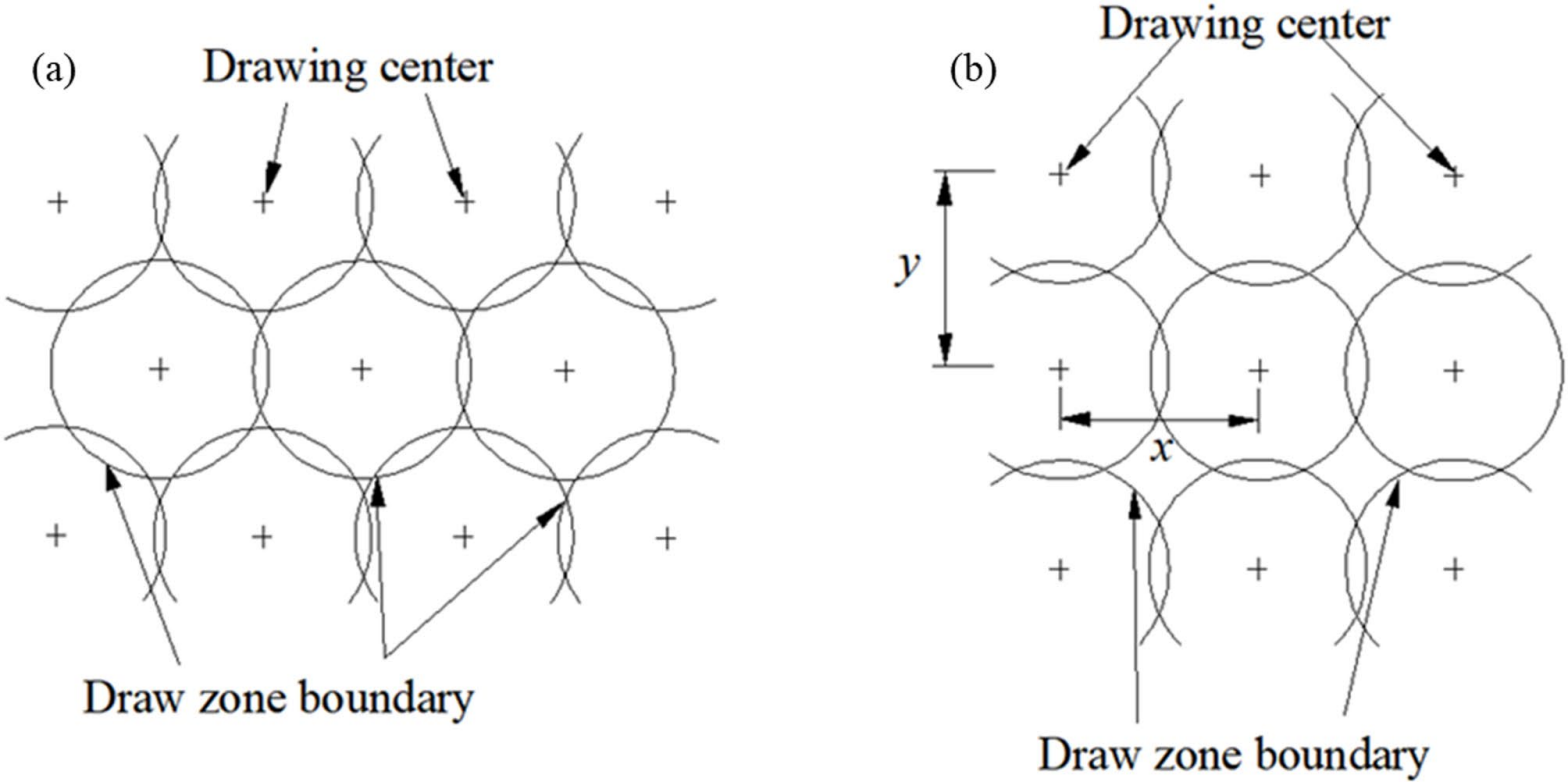
Idealized cross-section ore drawing point spacing (**a**) Hexagonal (**b**) Square.

In the design of the block caving mining method, ore drawing management and the stability of the bottom structure are the core factors ensuring production continuity and economic performance^25^. A stable bottom structure provides the physical foundation for planned and orderly ore drawing, while a scientific and rational control of drawing sequence and intensity can optimize the stress state of the bottom structure and maintain its integrity, thereby sustaining long-term extraction efficiency. Therefore, the essence of bottom structure design lies in achieving a balance among ore recovery rate, construction difficulty, and structural stability. From a geometric perspective, if the cross-section of a draw zone is simplified as a circle, its arrangement pattern directly influences resource recovery. Studies have shown that a regular hexagonal layout (Fig. 2(a)) can maximize coverage of the stope projection area and minimize rib losses between adjacent draw zones. In contrast, the traditional square layout (Fig. 2(b)) generates larger draw dead zones under the same spacing conditions, which is unfavorable for maximizing resource recovery^26^.

**Fig. 3:**
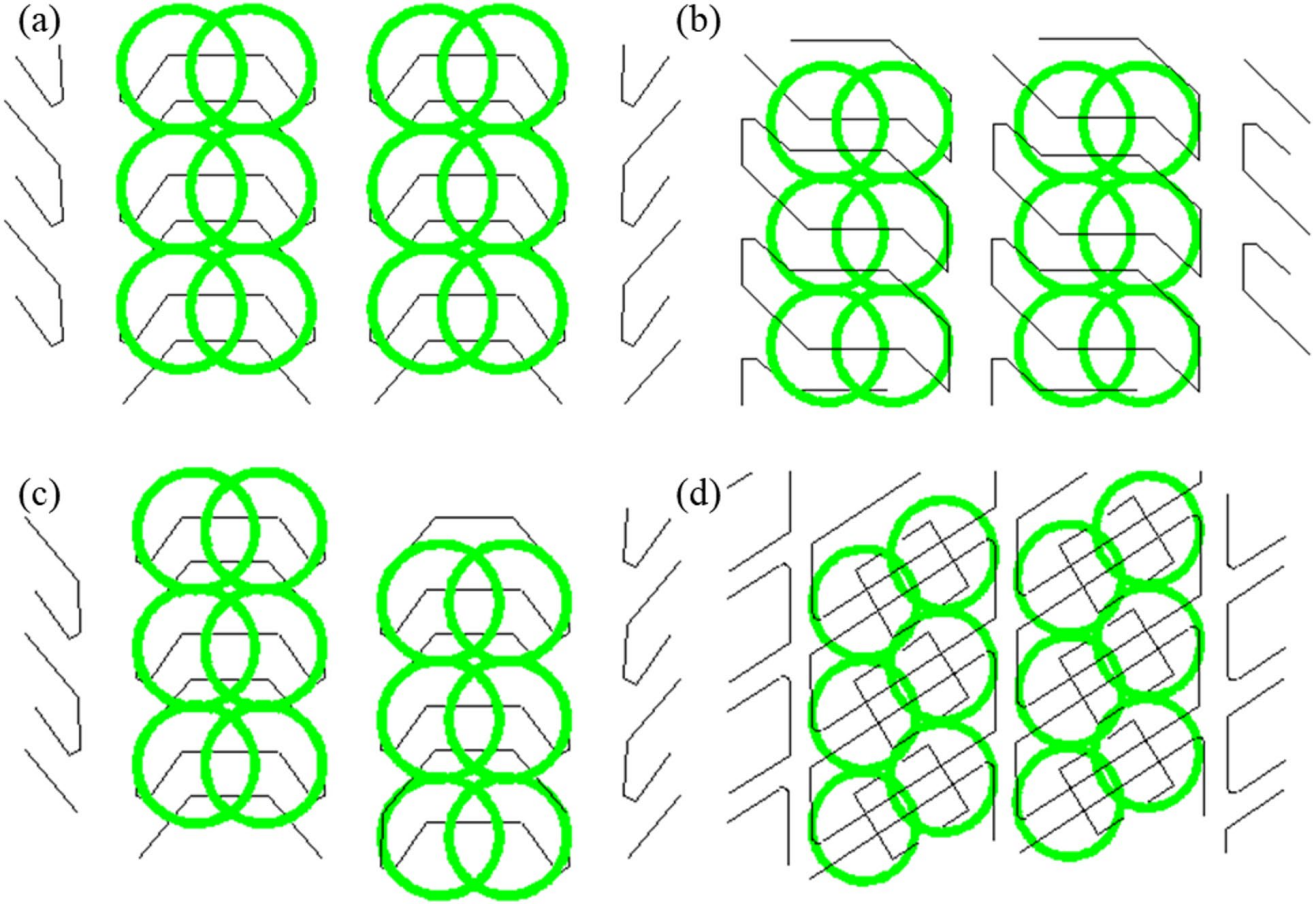
Idealized cross-sections of four main extraction-level layout types (**a**) Chevron (**b**) Henderson (Z-type) (**c**) Branch Chevron (**d**) Tennant type.

A comparison was conducted among four mainstream extraction-level layout schemes, as shown in Fig. 3. Under the condition that the diameter of the draw zones remains constant and adjacent zones just overlap at the secondary peak, numerical simulations indicate that paired drawpoints served by the same ore pass exhibit significant spatial overlap effects. Based on analyses of geometric coverage efficiency and structural stability indices, the Teniente and branching herringbone layouts demonstrate clear advantages in terms of adaptability to ore flow behavior^27^. However, considering the actual operational conditions of the Lower Orebody, the Teniente layout presents certain limitations for efficient ore extraction using electric LHDs due to roadway turning radius constraints and geometric restrictions. Taking into account equipment compatibility and production efficiency, the branching herringbone bottom structure not only maintains the advantages of high coverage efficiency and robust mechanical stability, but also features a roadway topology that better aligns with the travel paths and turning requirements of LHDs, demonstrating superior scientific rationality and engineering practicality. Therefore, the branching herringbone layout is selected for the bottom structure of the Lower Orebody to achieve optimal coordination between ore drawing efficiency and structural stability.

## Theoretical study on bottom structure parameters of the lower orebody

### Geometric relationships of bottom structure parameters

During block caving operations, the bottom structure is subjected to a complex dynamic stress environment induced by ore caving, draw-induced disturbances, and advancing undercutting. The configuration of its parameters directly affects both structural stability and ore drawing efficiency. The bottom structure consists of the extraction level and the undercut level, and its structural parameters can be categorized into vertical and planar parameters^28^. The vertical parameters mainly include the vertical distance between the extraction and undercut levels (H₁) and the undercut height (H₂). The planar parameters primarily include drawpoint crosscut spacing, undercut drift spacing, extraction drift spacing, and the upper and lower opening dimensions of the ore pass. The overall research framework follows the principle of “first satisfying ore drawing and production organization requirements, and then verifying structural stability.” Accordingly, the layout of drawpoints and the geometric parameters of the ore pass are determined first, after which the key vertical parameters affecting sill pillar stability are further optimized on this basis.

### Determination of the vertical distance (H₁) between the extraction level and the undercut level

The vertical distance (H₁) between the extraction level and the undercut level is a key parameter controlling sill pillar height and the safety of roadway engineering. It directly affects the load-bearing capacity of the gob-side (peach-shaped) pillar and the overall stability of the bottom structure. At the same time, it is constrained by factors such as construction methodology, drilling equipment capacity, resource recovery rate, service life, and mining depth. Through literature review and field investigation, the vertical distance data of representative block caving mines worldwide were compiled, as shown in Table 3^29–33^. The results indicate that the vertical distance generally ranges from 10 to 22 m.

**Table 3:** Examples of the vertical distance between the undercut level and the extraction level.

Mine name	Vertical distance between the undercut level and the extraction level–m
Palabora Lift1	18
Esmeralda	18
Henderson	18.3
Northparkes E26 Lift1	18
Northparkes E26 Lift2	14
Northparkes E48	22
New Afton Lift1	17
Argyle	15
Oyu Tolgoi Lift1	17
PTFI DMLZ	15
PTFI (Grasberg Block Cave)GBC	20
Tongkuangyu Copper Mine	10–15
Pulang Copper Mine	15–18

From the perspective of roadway protection, an appropriate increase in H₁ is beneficial for enhancing the sill pillar height and improving roadway stability. However, if the vertical distance is excessively large while the drawpoint crosscut spacing remains unchanged, a pointed pillar crown may form at the top of the pillar, resulting in limited improvement in overall stability. In terms of construction conditions, the excavation of the ore pass raise and the capacity of longhole drilling and charging operations directly constrain the feasible vertical distance. Considering the performance of the current drilling jumbos and charging equipment, the vertical distance between the extraction level and the undercut level is approximately 20 m. From the perspective of resource utilization, the sensitivity of recovery rate to sill pillar height varies with orebody occurrence. At the Tongkuangyu Copper Mine, sill pillar height has a significant impact on recovery efficiency. In contrast, for steeply dipping orebodies with large block heights, enlargement of the bottom structure has a relatively limited influence on recovery. Taking into account the service life of the block and the high in-situ stress conditions associated with deep mining, a moderate increase in H₁ can enhance the safety margin. Considering engineering safety, construction feasibility, and production organization requirements comprehensively, the vertical distance between the extraction level and the undercut level is reasonably determined to be approximately 20 m.

### Extraction-level structural parameters and hang-up control

#### Analysis of hang-ups and extraction-level structural parameters

The structural parameters of the extraction level are closely related to the dimensions of the ore pass, which directly influence the occurrence of hang-ups. In this study, five ore pass configurations were established, and hang-up prediction analyses were conducted using BCF software. For all schemes, the upper opening length was fixed at 15 m. The upper opening widths were set at 10 m, 15 m, 15 m, 12 m, and 15 m, respectively. The lower opening lengths were 15 m, 15 m, 12 m, 15 m, and 15 m, while the corresponding lower opening widths were 4.5 m, 4.5 m, 4.5 m, 5.0 m, and 5.0 m. Since BCF is highly sensitive to width variations, Schemes II and III, which share identical width parameters, are essentially equivalent; their hang-up prediction results are presented in Table 4. As shown in Table 4, when the upper opening length and width and the lower opening length remain constant, the lower opening width has the most significant effect on hang-ups. When the lower opening width increases from 4.5 m to 5.0 m, the hang-up rate under different draw heights decreases by 35%–86%. Therefore, the lower opening width is determined to be 5.0 m. The upper opening width mainly affects high-level hang-ups. Although the variation in hang-up rate is relatively small, to reduce the difficulty of handling high-level hang-ups, the upper opening width should not be less than 12 m. The relevant parameters were all determined based on previous engineering experience.

**Table 4:** Predicted bucket-jamming conditions under different ore-chute sizes.

Ore drawing height—m	Scheme I	Scheme II & Scheme III	Scheme IV	Scheme V
20	22.26%	21.65%	12.39%	14.43%
60	8.56%	8.36%	4.32%	4.85%
100	4.52%	4.18%	1.92%	1.74%
140	2.21%	2.00%	0.93%	0.84%
180	1.12%	1.09%	0.46%	0.52%
220	0.78%	0.68%	0.13%	0.11%

#### Comparative analysis

The structural parameters of the extraction level are influenced by multiple factors, including geological conditions, loading equipment, and production processes, and are directly related to production efficiency and ore drawing performance. Mines employing the block caving method typically determine optimal parameters through long-term operational experience. Table 5 lists the drawpoint crosscut spacing and loading drift spacing adopted in several block caving mines worldwide^34–37^. The branching herringbone layout is widely applied at the extraction level. As shown in the table, the spacing parameters of drawpoints are generally within the range of (14–17)×(15–22) m. Based on a comprehensive analysis of geological conditions, production experience, and hang-up prediction results, a preliminary range of extraction-level structural parameters can be determined. However, the final parameters should be established through project-specific optimization. In this study, the drawpoint crosscut spacing is ultimately selected as 28–36 m, and the extraction drift spacing is determined as 16–20 m.

**Table 5:** Structural parameters of extraction levels in domestic and overseas block caving mines.

Orebody name	Burial depth (m)	Main stress (MPa)	UCS (MPa)	MRMR	Spacing of extraction crosscuts (m)	Spacing of loading drifts (m)
NorthparkesE48	581	33	120	51–58	30	18
New Afton Lift1	600	30.2	60–71	45–52	27	15
Cadia East	1225	62.5	145–169	50	32	20
DMLZ	1400–1800	42.8–60	95–160	–	30	20
(Grasberg Block Cave) GBC	1200	32	5-140	60	30	20
New Afton B3 Oyu Tolgoi Lift1	760	34.8	–	68	27	16.5
	1300	–	40–70	40–45	31	18
New Afton C Zone	1150	46	–	–	27	18
El Teniente (Andes Norte)	–	55-60 MPa	–	–	34	22

### Selection of undercutting method and undercut cross-sectional form

The structural design of the undercut level is crucial for controlling the initial caving performance and ensuring the stability of the bottom structure^38^. The main undercutting methods include post-undercutting, pre-undercutting, and advancing undercutting^39^. Post-undercutting tends to induce stress concentration near the advancing front, resulting in high support requirements for roadways. Although pre-undercutting is beneficial for development safety, it may lead to ore compaction and increase the construction risk of ore passes, thereby slowing down the production ramp-up. In contrast, the advancing undercut method achieves a balance between stress environment improvement and production efficiency. It effectively reduces the risk of ground pressure damage in drawpoint crosscuts and is more suitable for mines under deep and complex stress conditions. Therefore, the advancing undercut method is adopted in this study. Regarding the cross-sectional form, several schemes—including ring fan drilling, high flat undercut, inclined narrow-section undercut, flat undercut, and trench undercut—were comparatively evaluated. Considering the large burial depth, high in-situ stress, and fractured surrounding rock conditions, the inclined narrow-section undercut is selected. This method requires less mucking volume, offers higher advance efficiency, and allows better control of the clamping effect, making it well suited to deep high-stress environments. In comparison, the ring fan drilling scheme involves extensive downward longhole drilling and is less favorable for construction; the trench undercut lacks support from small sill pillars; and the high flat undercut requires substantial initial investment. Therefore, these alternatives are not suitable for the conditions of this mine.

### Optimization of the undercut height (H₂)

The undercut height (H₂) directly influences the initial caving conditions, ore fragmentation, and undercutting efficiency. An appropriate increase in undercut height can enlarge the free surface and reduce the size of initial caved ore. For example, the E26 orebody at Northparkes Mine adopted a multi-level high undercut scheme to ensure early production capacity^40^. However, an excessively large undercut height increases the difficulty of longhole drilling, enlarges the workload, and leads to irregular roof profiles, thereby reducing operational efficiency and increasing construction time and costs. Data from block caving mines worldwide indicate that the undercut height generally ranges from 4 to 16 m^41^, and relatively lower heights can also satisfy production requirements. Considering the drilling equipment capacity of the mine, control of oversized blocks during the initial caving stage, and the risk of remnant pillar formation, it is noted that when the distance between the crown of the peach-shaped pillar and the caved ore boundary is less than 5 m, remnant rock pillars are likely to form and induce stress concentration. Therefore, the undercut height H₂ is ultimately determined to be 6 m, so as to balance caving performance, structural safety, and construction efficiency.

## Numerical simulation study and parameter determination of the bottom structure in the lower orebody

The stability of the bottom structure is significantly influenced by key parameters such as drawpoint crosscut spacing, extraction drift spacing, and the vertical distance between the extraction and undercut levels. To investigate the effects of these parameters on structural stability, a three-dimensional numerical model was established using FLAC3D. Through comparative analyses of multiple parameter combinations, the deformation characteristics, stress distribution, and evolution of plastic failure in the bottom structure were systematically examined. An orthogonal experimental design method was adopted for the numerical simulations. Three primary factors were considered: drawpoint crosscut spacing (28–36 m), extraction drift spacing (16–20 m), and the vertical distance between the extraction and undercut levels (18–22 m). Each factor was assigned three levels for combined analysis.

The calculation was conducted using the Mohr–Coulomb elastoplastic constitutive model based on the three-dimensional fast Lagrangian explicit finite difference method. The roadway excavation, floor cutting, and ore-pass excavation areas were simulated using null elements. The boundary and initialization conditions adopted during the modeling process were as follows:

1. The model boundaries were subjected to the in-situ stresses measured on site.
2. The top boundary of the model was subjected to the overburden load, the bottom boundary was fixed, and the lateral boundaries were constrained by horizontal displacement with axial displacement fixed.
3. The gravitational acceleration was set to 9.81 m–s², acting vertically downward.
4. By combining the displacement boundary conditions and the initial self-weight stress field, the program automatically solved the model until equilibrium was achieved.

To ensure the feasibility and reliability of the calculations, the numerical simulation analysis was conducted based on reasonable simplifications of the actual field conditions. According to the characteristics of the bottom structure, the following assumptions were adopted during the modeling process:

1. The influence of small-scale structural planes was neglected.
2. All rock strata were regarded as isotropic continuous media.
3. During the simulation, the effects of dynamic loads such as blasting-induced shock waves and seismic waves are neglected.

At the initial stage of the numerical calculation, the in-situ stress field was reproduced by applying the corresponding boundary conditions and initialization conditions.

### Development of the numerical simulation scheme and model construction for the bottom structure

#### Development of the numerical simulation scheme for bottom structure parameters

**Table 6:** Numerical simulation parameters of the bottom structure.

Spacing of extraction crosscuts (m)	Spacing of loading drifts (m)	Vertical distance between extraction level and undercut level (m)
28	16	18
32	18	20
36	20	22

Based on the previously determined ranges of extraction-level structural dimensions (drawpoint crosscut spacing × extraction drift spacing) and the vertical distance between the extraction and undercut levels, appropriate parameter levels were selected, as shown in Table 6. If a full-factorial experimental design were adopted, a three-factor, three-level scheme would theoretically require 27 simulation cases, resulting in an excessive workload^42^. To improve efficiency while ensuring result reliability, an orthogonal experimental design was employed. According to the orthogonal table arrangement, three factors (A, B, and C) with three levels (1, 2, and 3) were organized into nine simulation schemes, as presented in Table 7. The experimental design followed the principle of cross-combination under controlled variable conditions, ensuring systematic parameter pairing while significantly enhancing computational efficiency.

**Table 7:** Orthogonal numerical simulation schemes of the bottom structure.

Scheme No.	Extraction crosscut spacing A (m)	Loading drift spacing B (m)	Vertical distance between extraction and undercut levels C (m)
1	28 (1)	16 (1)	18 (1)
2	28 (1)	18 (2)	20 (2)
3	28 (1)	20 (3)	22 (3)
4	32 (2)	16 (1)	22 (3)
5	32 (2)	18 (2)	20 (2)
6	32 (2)	20 (3)	18 (1)
7	36 (3)	16 (1)	20 (2)
8	36 (3)	18 (2)	22 (3)
9	36 (3)	20 (3)	18 (1)

**Fig. 4:**
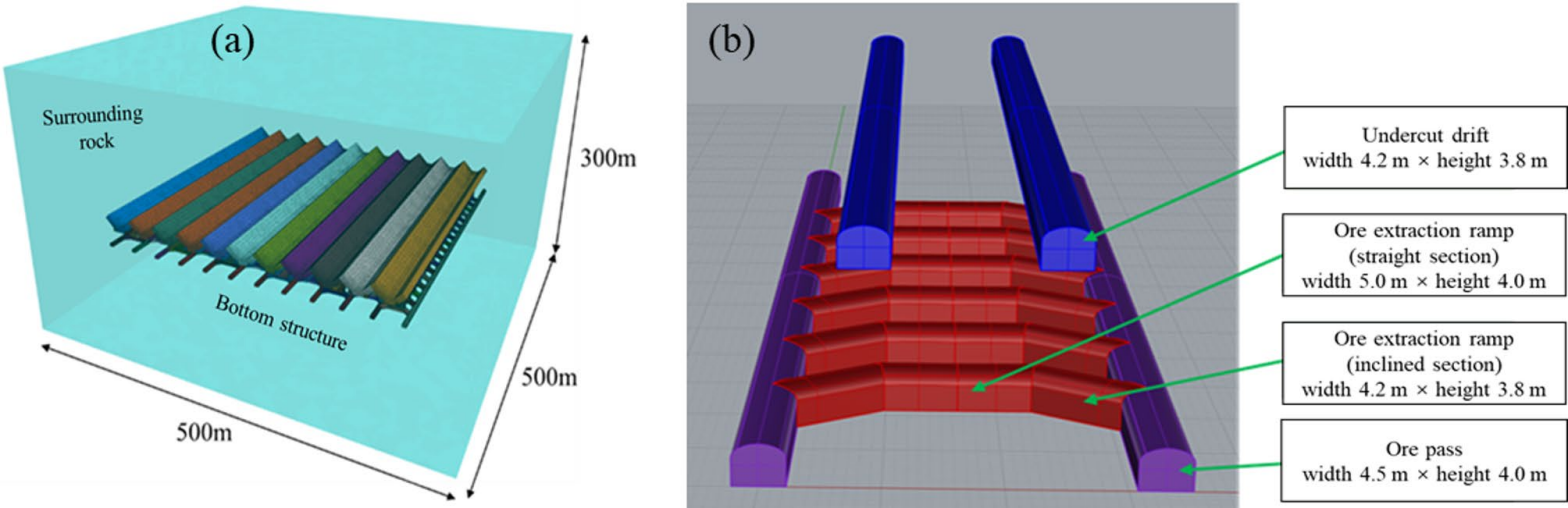
FLAC3D model (**a**) Model dimensions (**b**) Schematic of bottom structure drift cross-section dimensions.

Figure 4 illustrates the FLAC3D numerical model. Parameter-specific models were constructed according to Table 7, and RHINO was used to complete the pre-processing for FLAC3D computation. To fully capture the influence range of undercut excavation, the model core consists of 12 drawpoint crosscuts and 18 rows of extraction drifts. The model boundaries were extended outward to minimize boundary effects, resulting in an overall model size of 500 m × 500 m × 400 m. A locally refined mesh was adopted in the bottom structure zone, while relatively coarser grids were used in the outer regions. After optimization, the total number of elements in the model exceeded 6 million, as shown in Fig. 4(a). According to the combinations of extraction drift spacing and undercut drift spacing, different test schemes were established. The upper opening widths of the ore pass were set at 12 m, 14 m, and 16 m, and the corresponding upper opening lengths were 14 m, 16 m, and 18 m. In the numerical model, the clear cross-sections of the drawpoint crosscuts and the inclined sections of the extraction drifts were 4.5 m × 4.0 m; the clear cross-section of the ore pass section in the extraction drift was 5.0 m × 4.0 m; and the clear cross-section of the undercut level roadway was 4.2 m × 3.8 m. The extraction level layout adopted the branching herringbone configuration, as shown in Fig. 4(b).

#### Construction sequence of the bottom structure and initial in-situ stress equilibrium

##### Construction sequence of the bottom structure

**Fig. 5:**
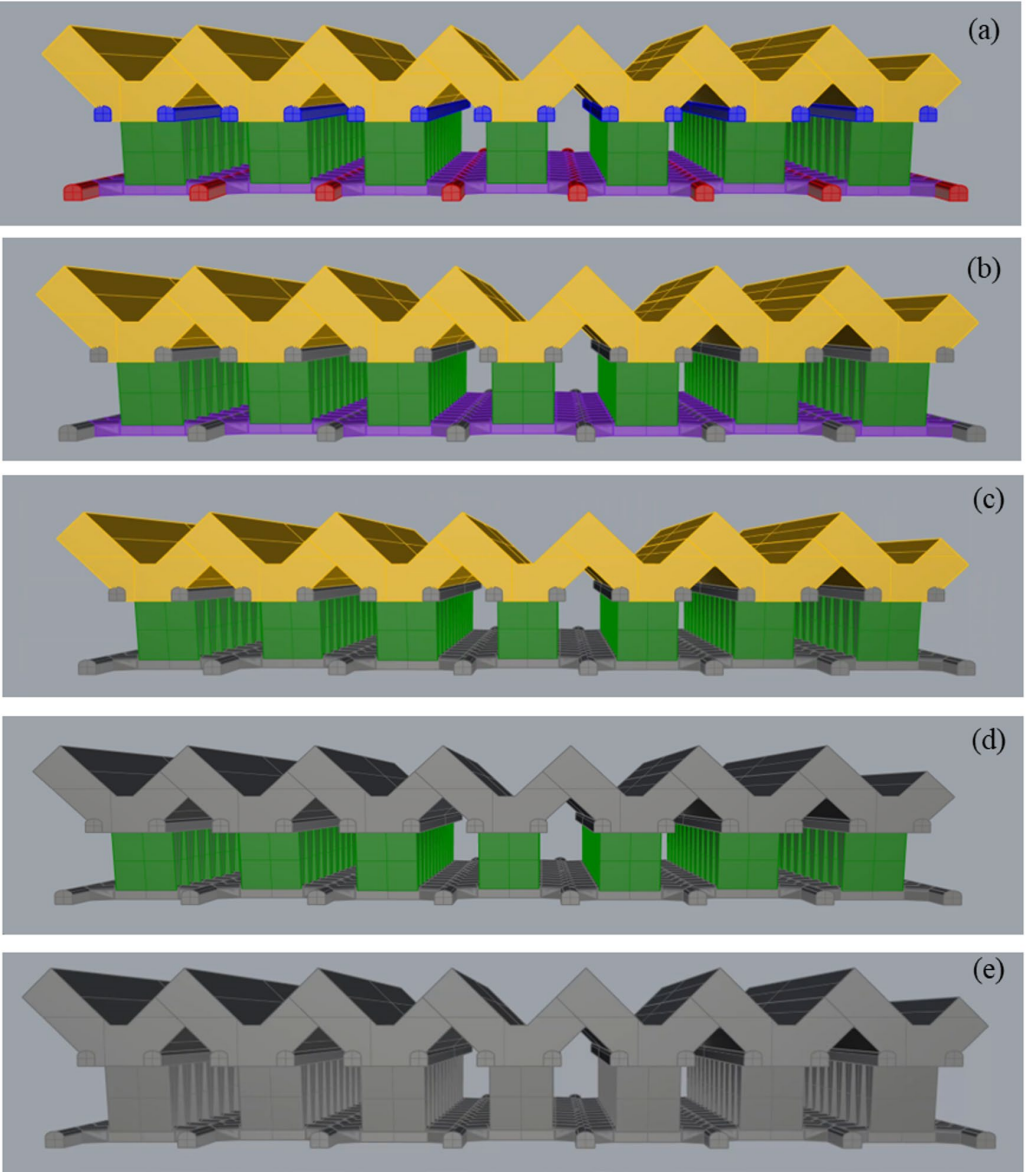
Simulation steps for bottom structure stability (**a**) Step 1 (**b**) Step 2 (**c**) Step 3 (**d**) Step 4. (**e**) Step 5.

Mining engineering involves complex rock mass mechanical responses, and the deformation and failure behavior are controlled not only by the current excavation state but also by the previous mining history and construction sequence^43^. To realistically reflect the mechanical evolution characteristics of the bottom structure during actual block caving operations in the Lower Orebody and to conduct parameter optimization research, a stepwise excavation numerical simulation scheme was established. The simulation process was divided into five stages, as illustrated in Fig. 5: Step 1: Initial state without excavation, with the model in equilibrium under the original in-situ stress field. Step 2: Completion of the excavation of drawpoint crosscuts and undercut drifts. Step 3: Completion of extraction drift excavation. Step 4: Completion of undercut excavation, ensuring that the effective undercut area is not less than the minimum area required for continuous caving. Step 5: Completion of ore pass excavation. Since the undercut structure is mainly located within the orebody and surrounding rock, different lithological groups were not introduced. Instead, unified rock mass mechanical parameters were adopted, corresponding to the andesite rock mass at depths of 1000–1200 m, as listed in Table 8.

**Table 8:** Mechanical parameters of ore and rock in the bottom structure model.

Parameter	Unit	Symbol	Value
Density	kg m^3^	density	2721
Elastic modulus	Pa	Young	7838e6
Poisson’s ratio	–	Poisson	0.26
Internal friction angle	°	friction	37.4
Cohesion	Pa	cohesion	3.714e6
Tensile strength	Pa	tension	1.503e6

##### Model boundary conditions and initial in-situ stress equilibrium

The − 800 m level was selected as the extraction level. The measured in-situ stress at this level is as follows: the minimum horizontal principal stress is 31.654 MPa, the maximum horizontal principal stress is 43.032 MPa, and the vertical principal stress is 31.457 MPa. Based on the measured in-situ stress data and the adopted mechanical parameters of the ore and surrounding rock, the stress boundary conditions applied at the top of the model and the lateral pressure coefficients were determined. Initial body stress gradients were applied in the X, Y, and Z directions in the model, with values of 0.0257 MPa–m, 0.0338 MPa–m, and 0.0269 MPa–m, respectively.

### Analysis of simulation results for the bottom structure construction process

#### Analysis of displacement characteristics

**Fig. 6:**
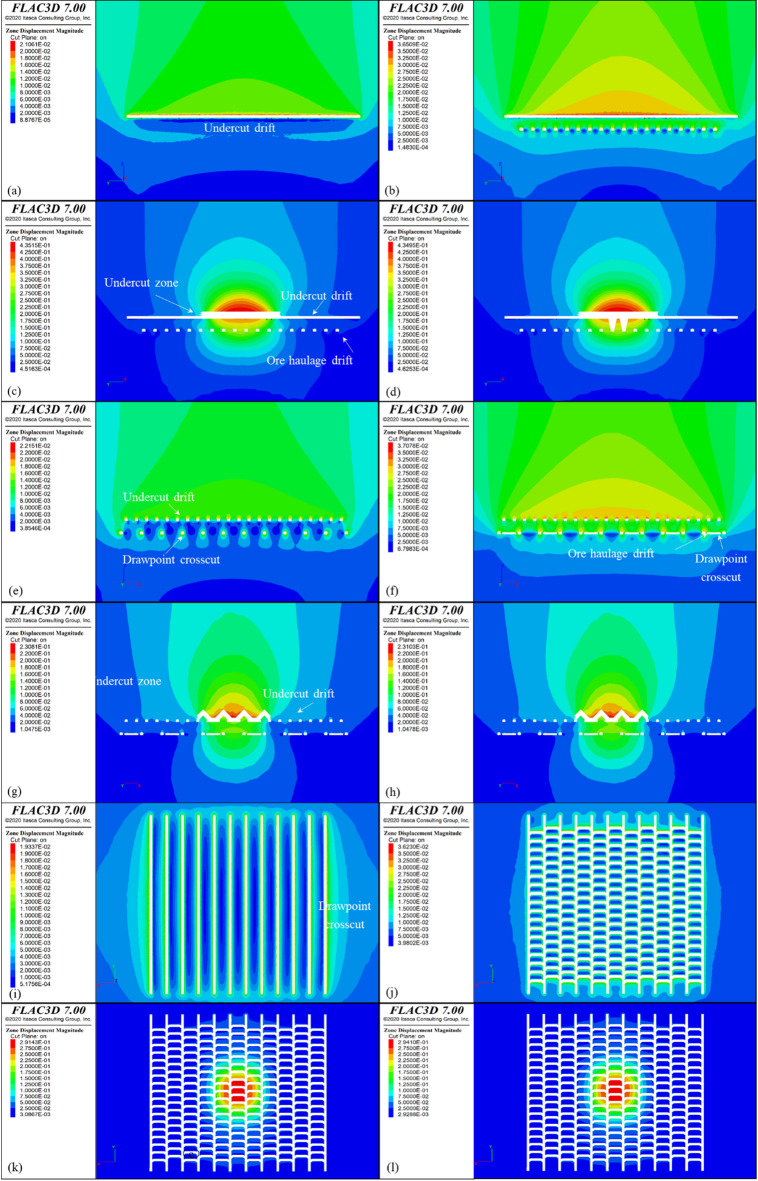
Displacement contour maps of surrounding rock in X, Y, and Z sections at different construction steps: (**a**–**d**) X-section (Steps 2–5); (**e**–**h**) Y-section (Steps 2–5); (**i**–**l**) Z-section (Steps 2–5).

A comparative analysis of the surrounding rock displacement distributions during the second, third, fourth, and fifth construction stages was conducted, as shown in Fig. 6. As construction progressed from Stage 2 to Stage 5, the displacement of the surrounding rock in the bottom structure exhibited an evolutionary pattern characterized by “slow initial growth, followed by a sharp increase, and finally a gradual stabilization.” After Stage 2, the overall displacement across all sections was relatively small. Upon completion of the extraction drift excavation in Stage 3, displacement became concentrated at the junctions between the extraction drifts and drawpoint crosscuts. After completion of the undercut excavation in Stage 4, displacement increased significantly, with high-displacement zones mainly distributed at the roof of the undercut space and at branching areas. Following the formation of the ore pass in Stage 5, only a slight additional increase in displacement was observed, with incremental deformation primarily concentrated near the lower opening of the ore pass and adjacent areas.

**Fig. 7:**
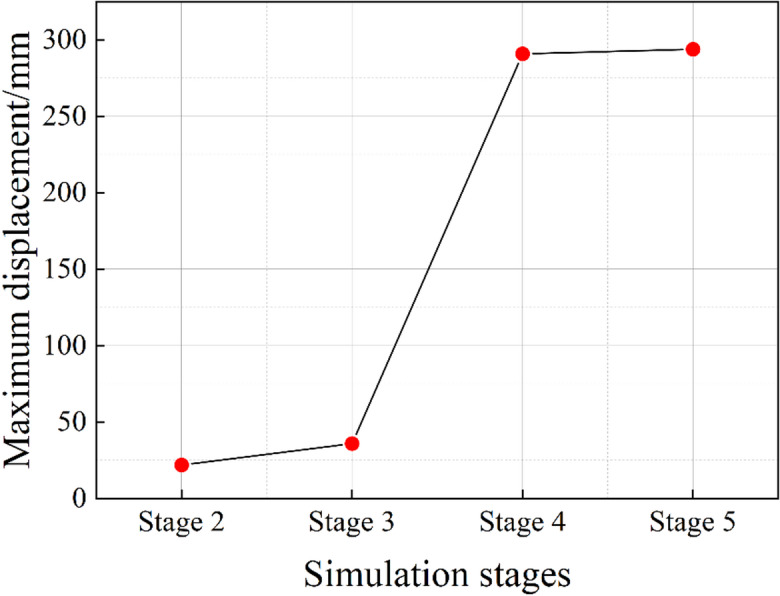
Variation of maximum displacement under different simulation stages.

The variation of maximum displacement under different construction steps is shown in Fig. 7. After completion of Stage 2, the overall displacement in the X, Y, and Z sections was relatively small, mainly concentrated at the floor and sidewalls of the drawpoint crosscuts and around the undercut drifts, with a maximum displacement of approximately 22 mm. Upon completion of Stage 3, displacement levels increased, and incremental displacement was concentrated at the junctions between extraction drifts and drawpoint crosscuts, forming localized displacement concentration zones, with a maximum displacement of approximately 36 mm. After completion of the undercut excavation in Stage 4, surrounding rock displacement increased markedly. High-displacement zones were distributed along the undercut space and above it. The maximum displacement reached approximately 291 mm, with about 200 mm observed at branching areas. The largest deformation occurred in the surrounding rock at the roof of the undercut space. Following completion of the ore pass in Stage 5, displacement in each section increased only slightly compared with Stage 4. The maximum displacement at the extraction level increased to approximately 294 mm, and displacement at branching areas increased to about 220 mm. The additional displacement increment was mainly concentrated near the lower opening of the ore pass.

#### Analysis of minimum principal stress characteristics

**Fig. 8:**
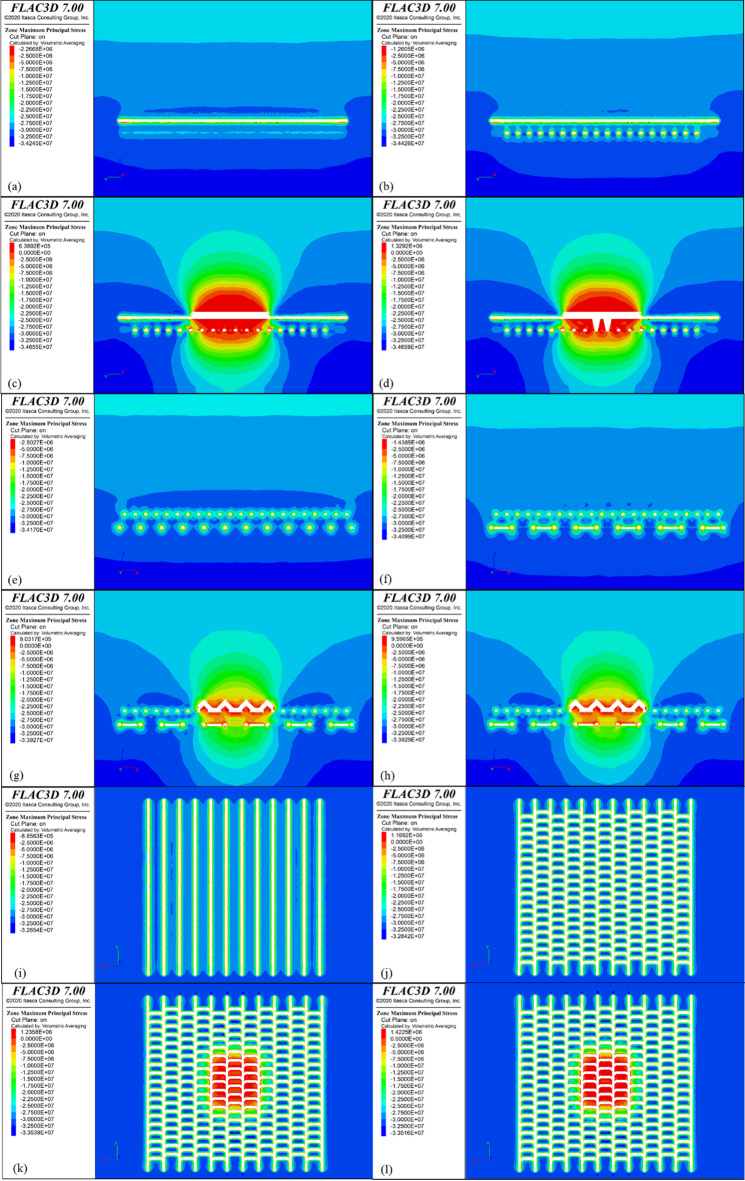
Minimum principal stress contour maps in X, Y, and Z sections at different construction steps: (**a**–**d**) X-section (Steps 2–5); (**e**–**h**) Y-section (Steps 2–5); (**i**–**l**) Z-section (Steps 2–5).

A comparative analysis of the minimum principal stress distribution during the second, third, fourth, and fifth construction stages was conducted, as shown in Fig. 8. With the progression of bottom structure excavation, the minimum principal stress state of the surrounding rock exhibits an evolutionary trend characterized by initial dominance of compressive stress, followed by the emergence of localized tensile stress, and eventual concentration of tensile stress in later stages.

**Fig. 9:**
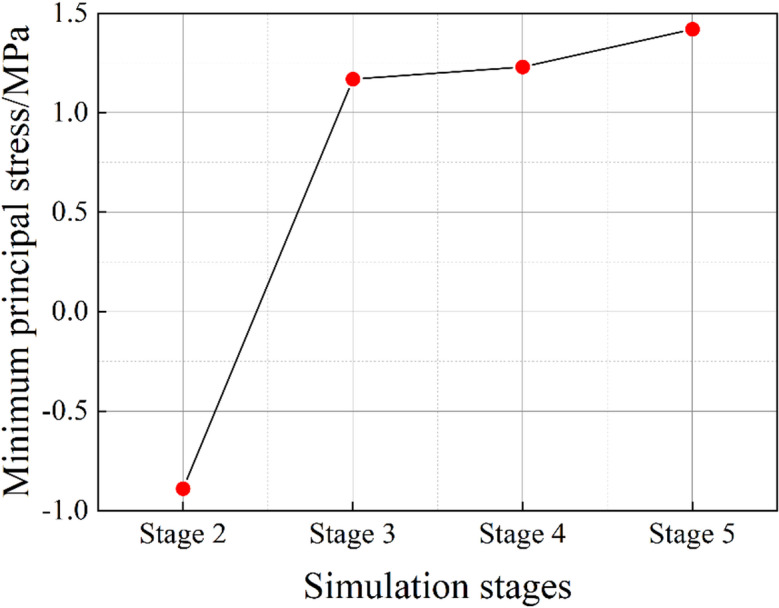
The variation of minimum principal stress under different simulation stages.

The variation of minimum principal stress under different construction steps is shown in Fig. 9. After completion of Stage 2, the surrounding rock is generally in a compressive stress state, with a minimum principal stress of approximately − 0.89 MPa, which is lower than the shear strength and uniaxial compressive strength of the rock mass, indicating overall stability. Upon completion of Stage 3, when the extraction drifts are formed, localized positive values of minimum principal stress begin to appear at branching areas, reaching a maximum of approximately 1.169 MPa. This suggests tensile stress concentration in local rock pillars and potential instability risk. After completion of the undercut excavation in Stage 4, the tensile stress zone expands significantly. Compressive stress decreases at the upper, middle, and lower parts of the structure, and a large tensile stress zone forms in the central portion of the extraction level. The minimum principal stress reaches 1.23 MPa, and the tensile stress at both ends of the undercut void reaches up to 0.64 MPa, which may adversely affect the stability of the surrounding rock in the undercut drifts. Following the formation of the ore pass in Stage 5, the minimum principal stress becomes further concentrated within the bottom structure. Tensile stress at the branching areas increases to 1.42 MPa, and the maximum tensile stress occurs at the extraction level, approaching the tensile strength of the rock mass. This indicates an increased risk of local failure and highlights the need for focused attention in subsequent support design. This indicates an increased risk of local failure and highlights the need for focused attention in subsequent support design.

In the X, Y, and Z sections, after Stage 2, the surrounding rock is dominated by compressive stress, with maximum compressive stress concentrated at the roof and sidewalls, and the rock mass remains stable. After Stage 3 excavation, localized tensile stress becomes evident in the branching zones, and positive minimum principal stress values appear. After completion of the undercut in Stage 4, high-magnitude tensile stress zones appear in all X, Y, and Z sections, particularly concentrated at the roof of the undercut space and at branching areas. After Stage 5, tensile stress becomes further concentrated locally, mainly near the lower opening of the ore pass and along the extraction level, with local minimum principal stress values approaching the tensile strength of the rock mass. Overall, stress redistribution during construction leads to localized tensile stress concentration, reducing the stability of certain surrounding rock zones and increasing the risk of tensile failure.

#### Analysis of plastic failure zones

**Fig. 10:**
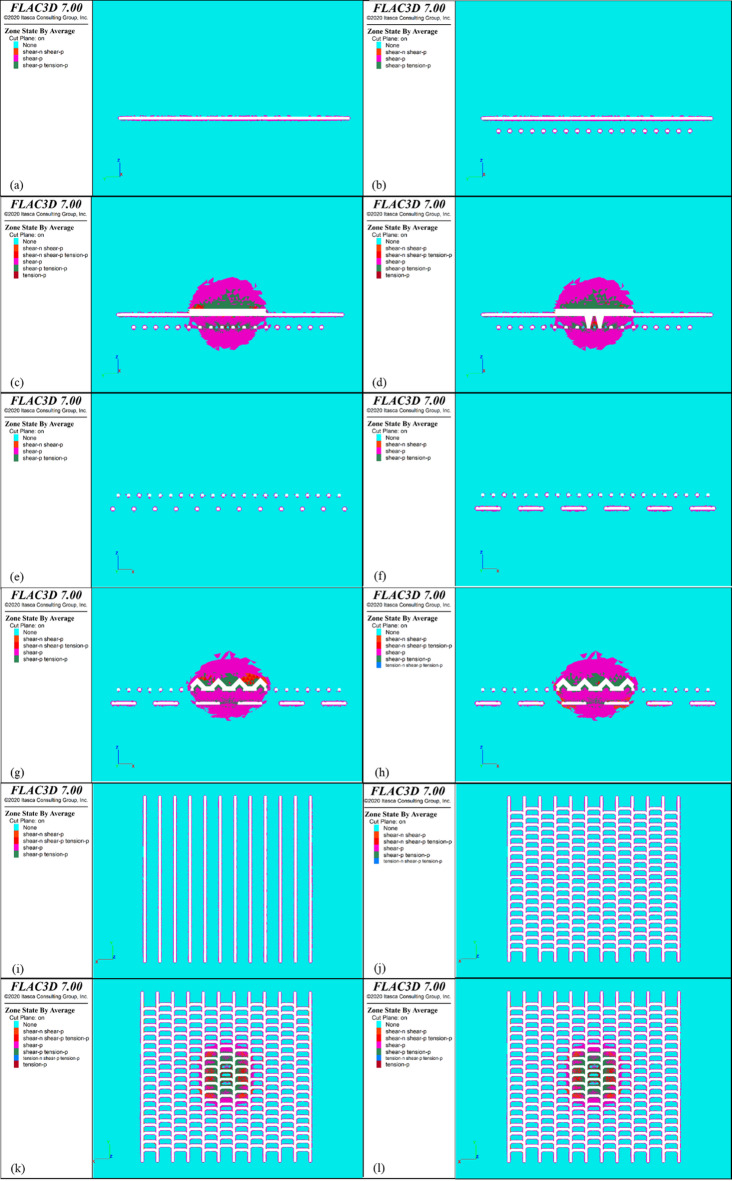
Plastic failure zone distributions in X, Y, and Z sections at different construction steps: (**a**–**d**) X-section (Steps 2–5); (**e**–**h**) Y-section (Steps 2–5); (**i**–**l**) Z-section (Steps 2–5).

A comparative analysis of the plastic failure zones during the second, third, fourth, and fifth construction stages was carried out, as shown in Fig. 10. During the construction of the bottom structure, stress redistribution within the surrounding rock leads to energy accumulation and release, resulting in progressive plastic failure of the rock mass. From Stage 2 to Stage 5, the plastic zones exhibit an evolutionary pattern characterized by “shallow localized failure-coalescence at branching areas-expansion around the undercut void-concentrated shear failure near the ore pass.”

**Fig. 11:**
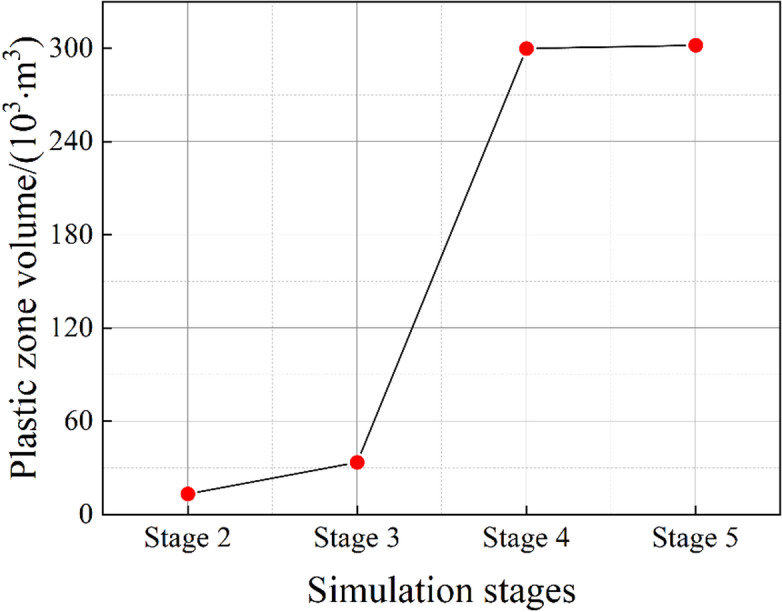
The variation of plastic zone volume under different simulation stages.

The variation of plastic zone volume under different construction steps is shown in Fig. 11. After completion of Stage 2, shallow plastic zones appear around the drawpoint crosscuts and undercut drifts, mainly confined to approximately 1 m from the excavation boundary. The volume of plastic zone is 13,251 m^3^. The deeper surrounding rock remains elastic, and the overall structure is stable. Upon completion of Stage 3, when the extraction drifts are formed, the plastic zone at the extraction level expands significantly, and plastic zones at the branching areas become interconnected. The dominant failure mode is a combination of tensile and shear failure The volume of plastic zone is 33,568 m^3^. After completion of the undercut excavation in Stage 4, the plastic zone further expands in the upper part of the undercut void and within the peach-shaped sill pillar. The plastic zones surrounding the drawpoint crosscuts and extraction drifts also extend noticeably. Plastic failure at the branching areas becomes fully connected, exhibiting combined shear–tensile failure, which facilitates ore and rock caving The volume of plastic zone is 300,012 m^3^. Following the formation of the ore pass in Stage 5, the plastic zone within the peach-shaped pillar is dominated by shear failure The volume of plastic zone is 302,180 m^3^. The plastic zones around the extraction level roadways and branching areas become fully interconnected, indicating potential local stability risks.

In the X, Y, and Z sections, after Stage 2, the plastic zones are limited to the immediate vicinity of the excavation boundaries, and overall stability is maintained. After Stage 3 excavation, the plastic zone at the branching areas expands significantly and becomes interconnected. After completion of the undercut in Stage 4, the high-displacement zones and plastic zones above the undercut void enlarge markedly, and the plastic zones at the branching areas coalesce, with shear–tensile composite failure as the dominant mode. After Stage 5, the plastic zones become more concentrated, mainly near the lower opening of the ore pass and within the peach-shaped pillar. Shear failure predominates, and the plastic zones around the extraction level roadways and branching areas become fully connected, indicating potential stability risks in the surrounding rock.

### Selection and determination of bottom structure parameters

Based on the comprehensive findings of the preceding analyses, the optimal design scheme for the bottom structure has been determined. First, according to theoretical analysis, numerical simulation results, and engineering analogy, the reasonable spacing of drawpoint crosscuts is identified as 32 m; therefore, the final drawpoint crosscut spacing is set at 32 m. Second, regarding the optimization of extraction drift spacing, factors including hang-up control, equipment compatibility, and structural stability were comprehensively considered. The reasonable spacing is determined to be 18 m, and thus the extraction drift spacing is finalized at 18 m. Finally, the selection of the vertical distance between the extraction and undercut levels is critical under deep, high-stress conditions, as it significantly affects structural stability and construction feasibility. The results indicate that when the vertical distance is 20 m, a large peach-shaped pillar with a height of 6.8 m can be formed, effectively meeting roadway protection requirements and significantly enhancing structural stability in a high-stress environment. In addition, this vertical distance is well matched with the effective drilling depth of medium- to long-hole drilling jumbos, avoiding the reduction in drilling quality associated with excessively deep boreholes. Although numerical simulations show that a 22 m vertical distance results in the smallest plastic zone volume, considering factors such as pillar recovery rate and equipment limitations, 20 m is preferred.

In summary, the optimal bottom structure parameters are determined as follows: drawpoint crosscut spacing of 32 m, extraction drift spacing of 18 m, and a vertical distance of 20 m between the extraction and undercut levels.

### Study on the advanced undercut distance

#### Analysis of the single-step undercut advance distance

The present undercut operation adopts upward fan-shaped medium-length hole blasting, similar to squeeze blasting in block caving without sill pillars. Since there is no overlying cover above the undercut level and the blasted ore is relatively loose with some compensation space available at the outlet, the blasting advance per round can be slightly greater than that used in conventional block caving without sill pillars. In this study, each undercut blasting round involves three rows of holes. The undercut blasting is conducted using underground medium-length hole blasting. The main drilling and blasting parameters include blasthole diameter (D), minimum burden (W), hole spacing (b), bottom spacing (a), and hole depth (L). A Simba 1354 drilling jumbo is employed to drill upward fan-shaped holes within the undercut drifts, with hole diameters ranging from *φ*76 to *φ*89 mm. Considering the actual mine conditions, the blasthole diameter is ultimately determined as φ76 mm. The minimum burden values currently adopted in the mine are listed in Table 9.

**Table 9:** Relationship between minimum resistance line and borehole diameter.

D (mm)	W (m)	D (mm)	W (m)
50–60	1.2–1.6	70–80	1.8–2.5
60–70	1.5–2.0	90–120	2.5–4

#### Numerical simulation scheme for advanced undercutting

**Fig. 12:**
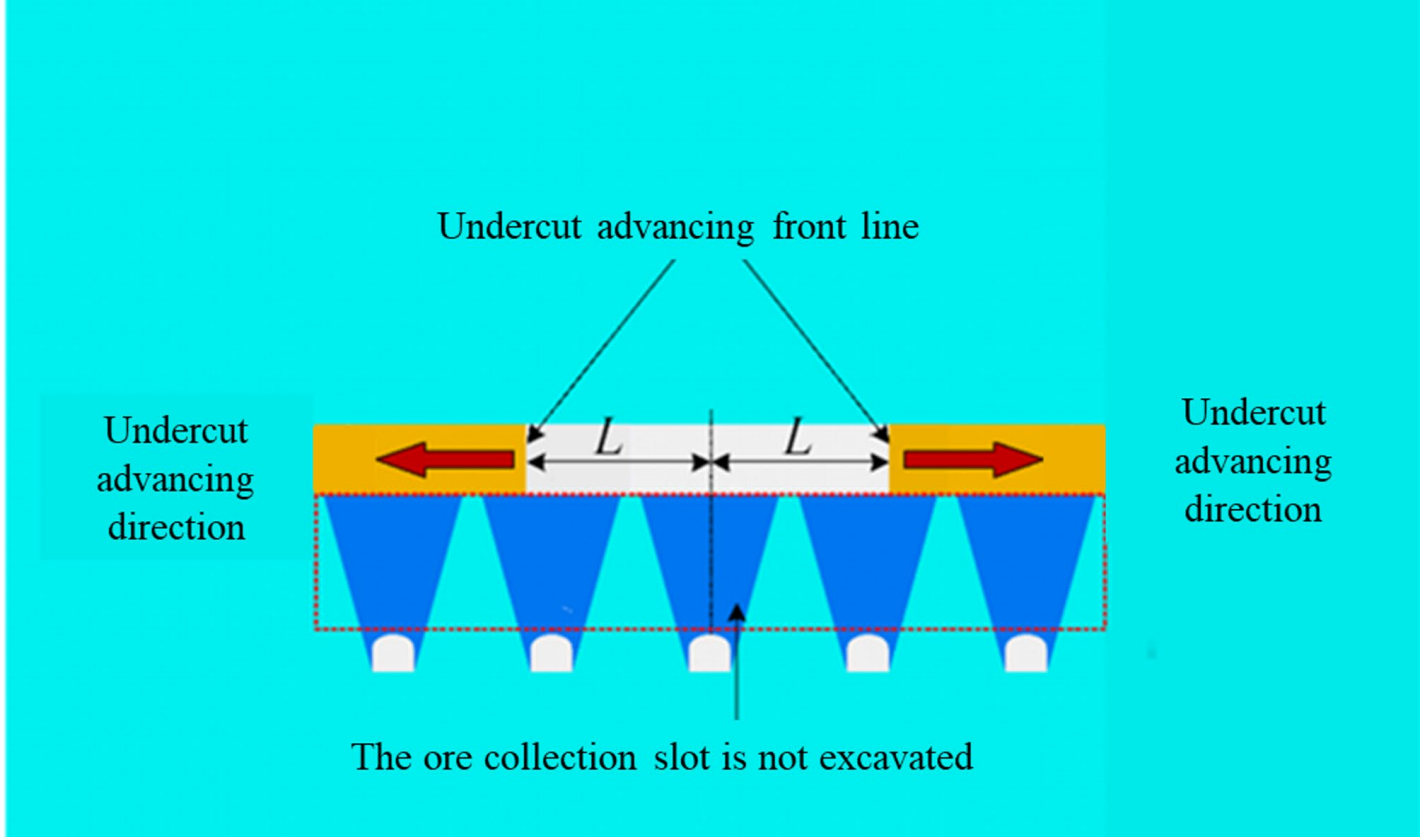
Schematic of progressive undercutting simulation.

**Fig. 13:**
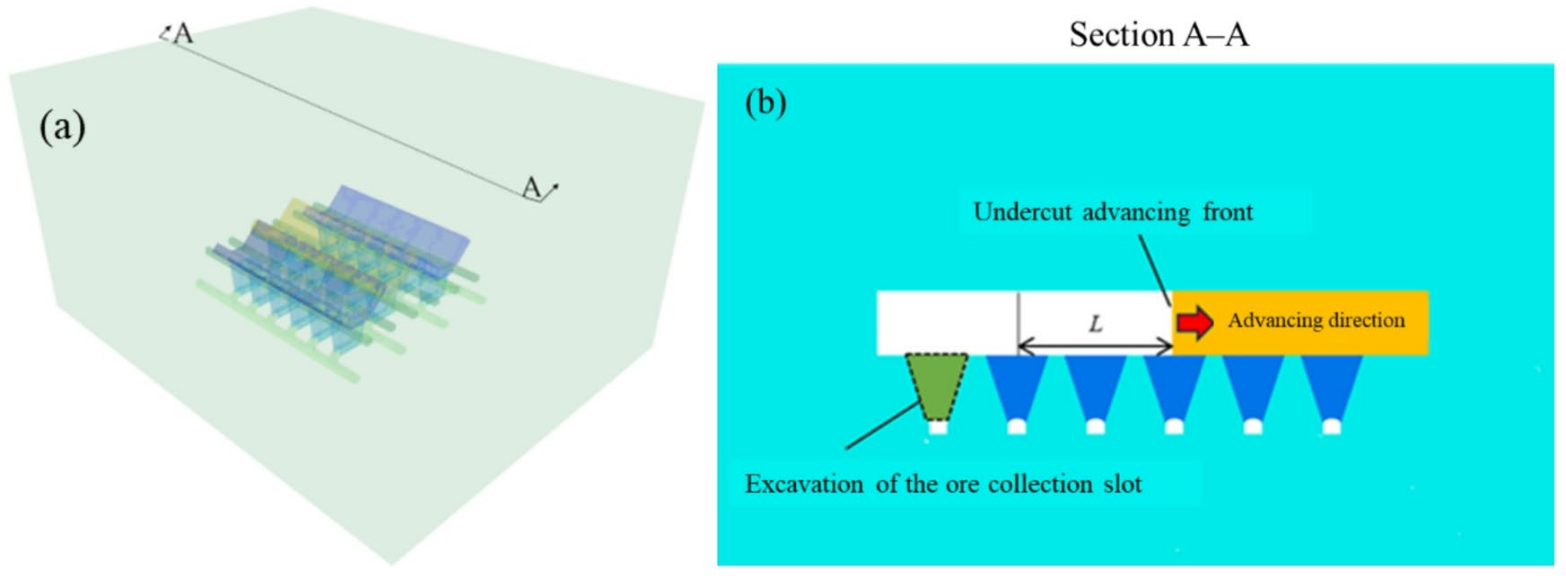
Dynamic simulation of progressive undercutting (**a**) Model schematic (**b**) Schematic of undercut advancement and ore chute construction.

Based on the established bottom structure parameters—vertical distance between the undercut and extraction levels of 20 m, drawpoint crosscut spacing of 32 m, extraction drift spacing of 16 m, undercut height of 6 m, ore pass upper opening dimensions of 12 × 16 m, and lower opening dimensions of 5 × 16 m—a numerical model was developed to investigate two categories of working conditions. Category I: Undercutting without excavation of the ore pass. In this case, the undercut advance distances were set to 12 m, 24 m, 36 m, 48 m, and 60 m, respectively. The ore pass beneath the undercut level was not excavated. The variation in stress concentration within the ore pass region under different undercut advance distances was analyzed, as shown in Fig. 12. Category II: Conditions with the ore pass already formed. The stress state at the location of the planned ore pass excavation was analyzed, as illustrated in Fig. 13. The relative distance (L) between the advancing undercut front and the ore pass front was used as the control parameter. The undercut was advanced in increments of 6 m per round. The simulation sequence first involved excavation of the drawpoint crosscuts, extraction drifts, and undercut drifts, followed by stepwise undercut advancement. The ore pass was then excavated according to the specified relative distance, thereby forming a dynamic construction simulation process.

#### Simulation analysis of advanced undercut distance with bilateral advancement

**Fig. 14:**
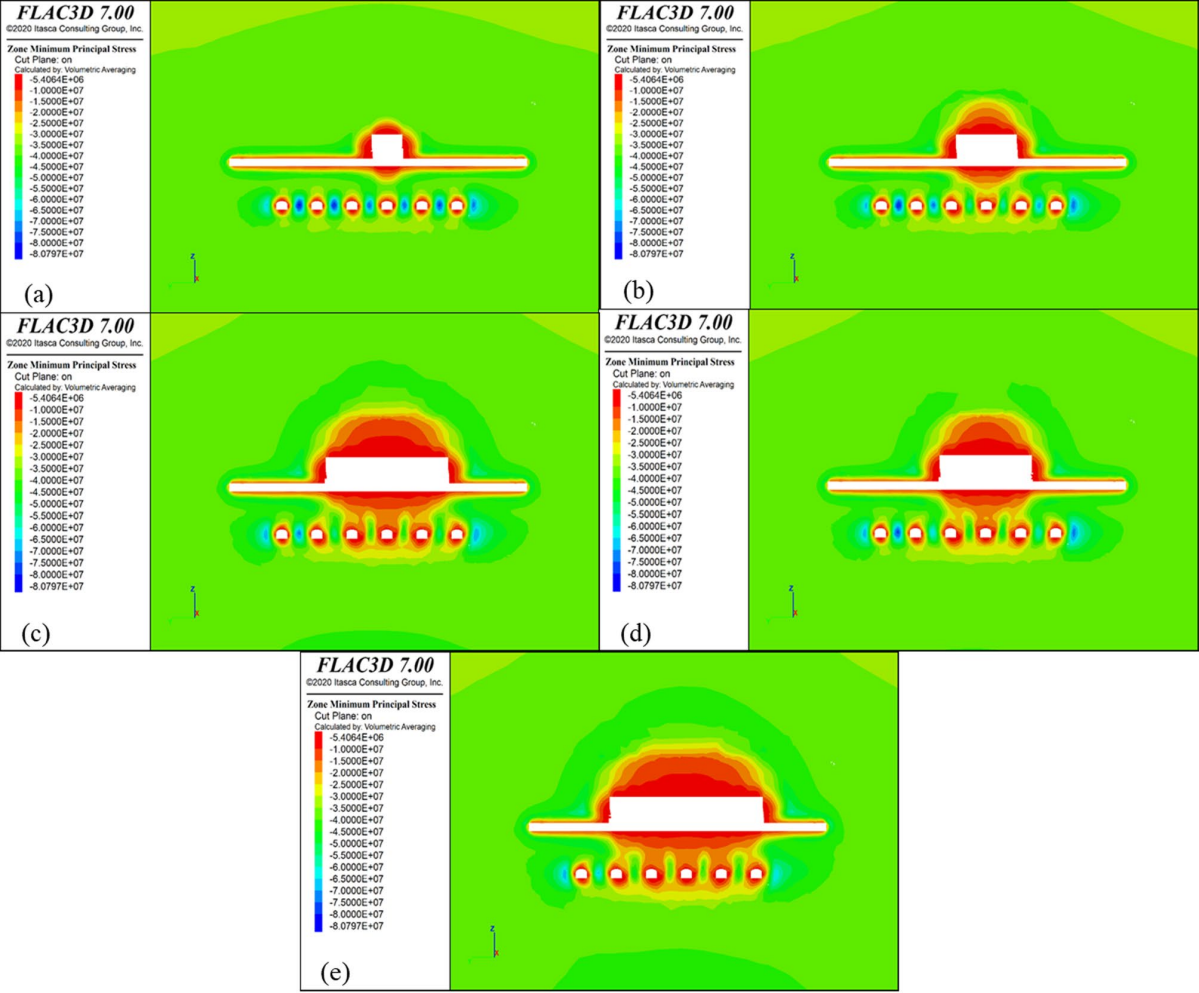
Maximum principal stress contour maps under different undercut distances: (**a**) 12 m (**b**) 24 m (**c**) 36 m (**d**) 48 m (**e**) 60 m.

Taking the maximum principal stress distribution of the surrounding rock as the evaluation criterion, the stress state of the ore pass under different advanced undercut distances was analyzed, as shown in Fig. 14. The results indicate that when the undercut advance distance is 12 m, the area surrounding the ore pass remains within a compressive stress concentration zone. When the undercut distance increases to 36 m, stress concentration is significantly alleviated, with only localized concentration remaining at the roadway roof. When the undercut distance reaches 60 m, stress is further released, and the concentrated zone at the roof is markedly reduced. These findings suggest that appropriately increasing the undercut advance distance can effectively improve the stress environment around the ore pass and reduce the risk of instability during excavation.

**Fig. 15:**
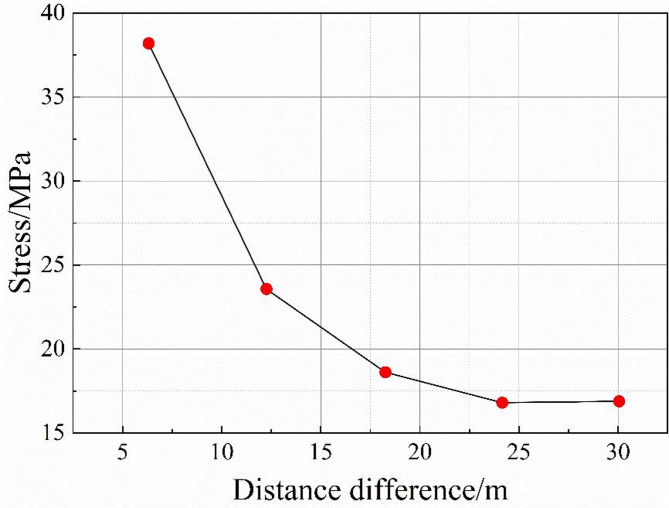
Stress distribution of surrounding rock between undercut and extraction levels.

Figure 15 illustrates the variation trend of stress within the ore pass under different relative distances between the ore pass and the advancing undercut front. The results show that once undercutting begins, the absolute stress value within the ore pass decreases significantly. With increasing undercut distance, the stress peak decays rapidly. When the undercut advances to 12 m, the stress within the ore pass reaches approximately 38 MPa, which is unfavorable for ore pass stability. When the undercut advances to 60 m, corresponding to a relative distance of 30 m, the stress within the ore pass tends to stabilize. Overall, the larger the undercut space, the weaker the stress concentration effect on the ore pass. Although this condition corresponds to the ideal scenario in which the ore pass has not yet been excavated, it still provides a valuable reference for determining the initial layout position of the first ore pass during early-stage bottom structure construction.

#### Simulation analysis of advanced undercut distance with unilateral advancement

**Fig. 16:**
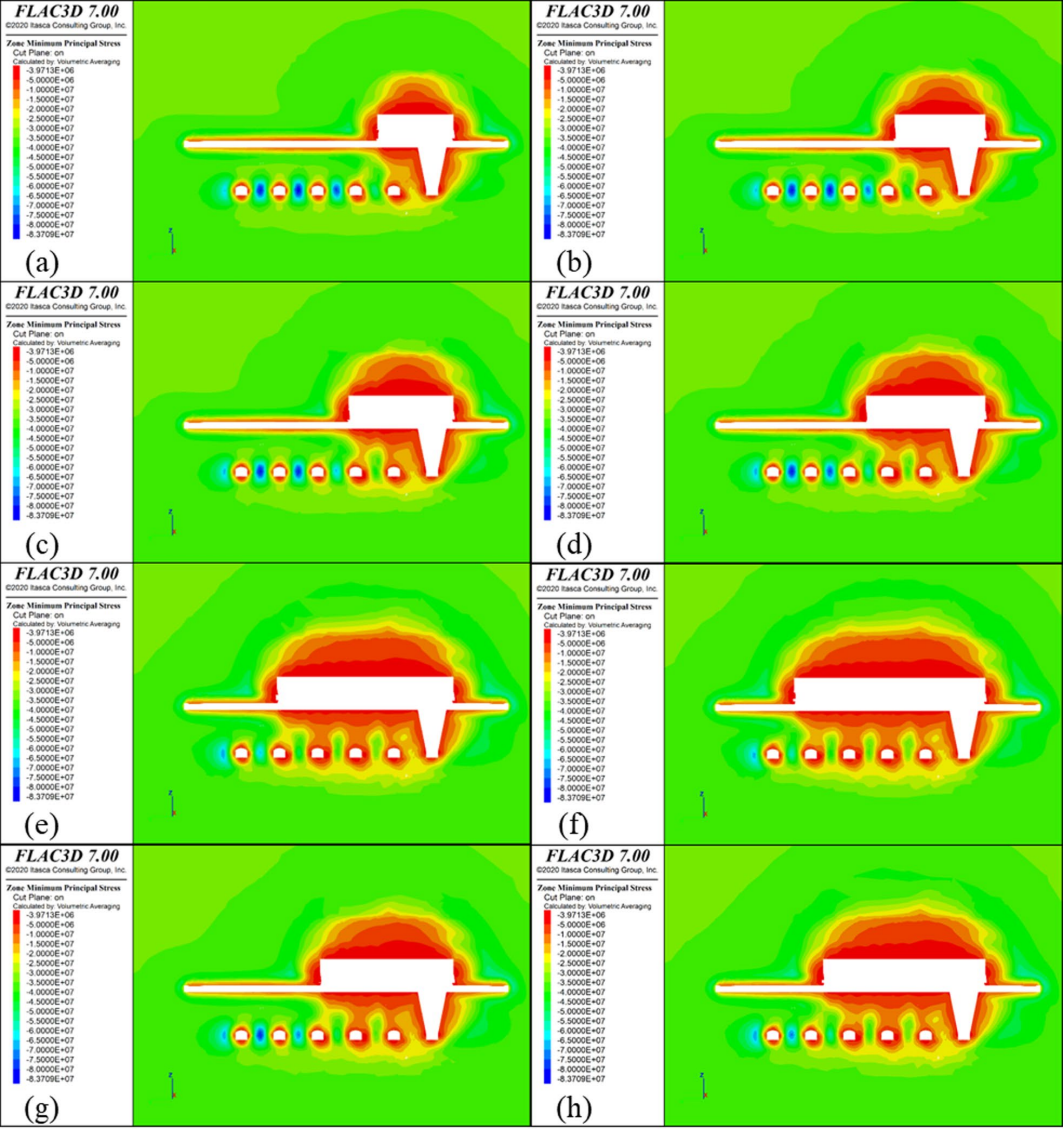
Minimum principal stress distribution contour maps after undercutting at 6.0 m–step and excavation of ore chutes (**a**) 6 m (**b**) 12 m (**c**) 18 m (**d**) 24 m (**e**) 30 m (**f**) 36 m (**g**) 42 m (**h**) 48 m.

During bottom structure construction, the advancing undercut method can effectively prevent the ore pass from being located within a stress concentration zone^44,45^. In this study, continuous undercutting was carried out with an advance step of 6.0 m per cycle to analyze the influence of the relative distance between the ore pass and the advancing undercut front on stress concentration. Figure 16 presents the Y-direction section of the minimum principal stress under different advanced undercut distances. The results indicate that after one or more ore passes have been excavated, the stress concentration within the next planned ore pass is significantly lower than that under the condition where no ore pass has yet been excavated. In addition, the accumulated strain energy within the rock mass is reduced, which is beneficial for construction safety.

**Fig. 17:**
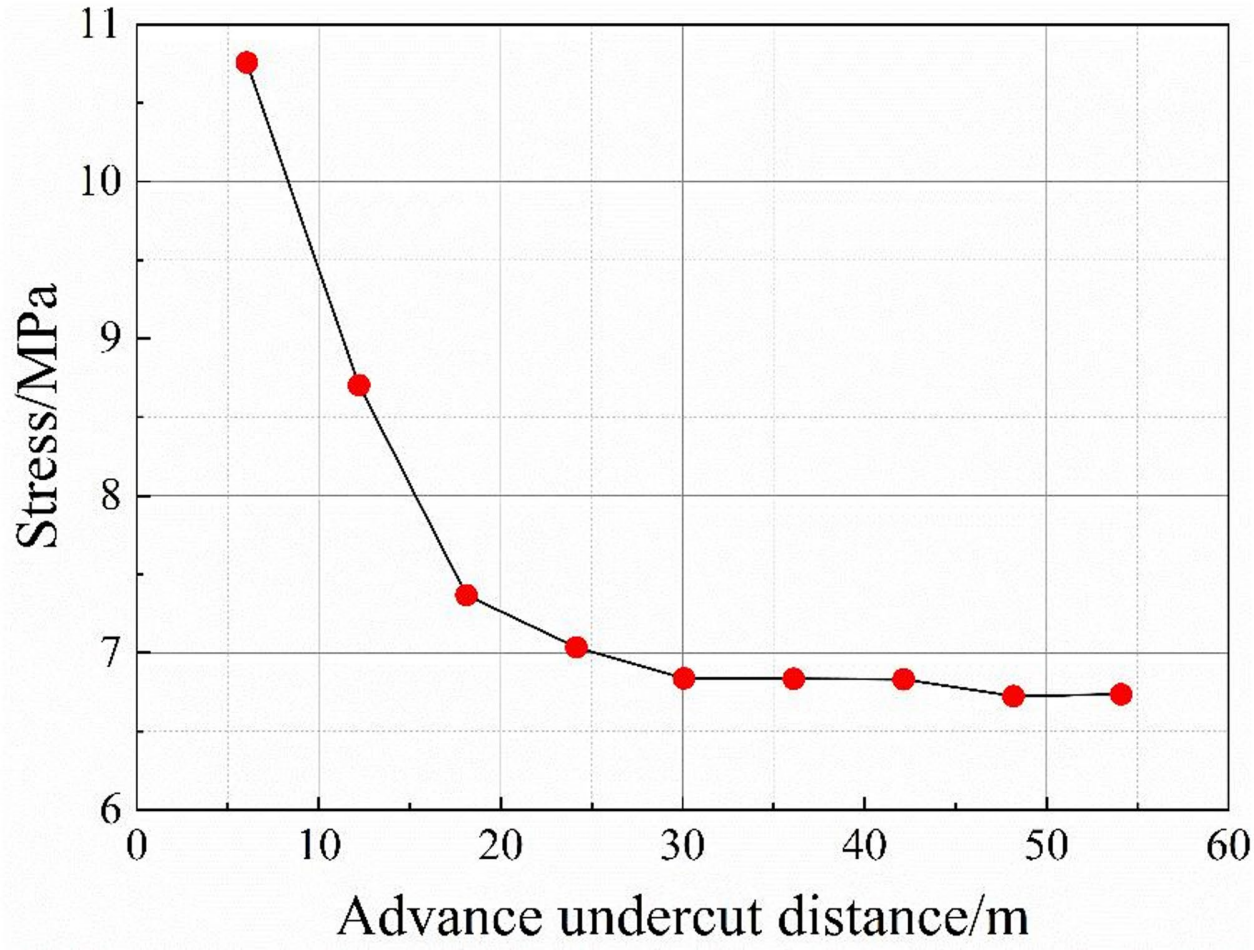
Relationship between advance undercut distance and concentrated stress in ore chutes.

As shown in Fig. 17, the concentrated stress within the ore pass decreases continuously with increasing advanced undercut distance. When the distance is less than 30 m, the stress variation is highly sensitive to the undercut advance. When the distance exceeds 30 m, the rate of stress reduction becomes more gradual. Once the distance exceeds 36 m, the stress generally falls below 6.86 MPa, reaching approximately 6.85 MPa at 42 m. Considering both construction safety and the requirement for timely ore production, this study recommends an advanced undercut distance of 30–36 m.

### Sensitivity analysis of the influence of various factors

Sensitivity analysis methods for orthogonal experimental design mainly include range analysis and variance analysis, which are two important approaches for evaluating the results of orthogonal experiments. Range analysis, also known as R-value analysis, quantifies the relative influence of different factors by calculating the range of each factor. It provides a direct and intuitive way to determine the sensitivity ranking of influencing factors. Compared with variance analysis, which requires strict assumptions regarding data distribution and experimental errors, range analysis does not require the introduction of blank columns or repeated trials. It can be directly applied based on numerical simulation results to evaluate sensitivity indicators. This makes it particularly suitable for geotechnical engineering problems characterized by non-uniform and nonlinear numerical models. Therefore, in this study, the range analysis method is adopted to perform sensitivity analysis on the orthogonal experimental results.

In field engineering, the magnitude of roadway deformation directly reflects the stability of the surrounding rock mass. Meanwhile, in numerical simulations, compared with the stress values within the rock mass, the volume of the plastic zone in the bottom structure more directly indicates the stability of the bottom structure. Therefore, in this study, roadway displacement and the plastic zone volume of the bottom structure were selected as the key indicators for evaluating bottom structure stability. The range analysis method was employed to calculate the range of the experimental indicators corresponding to different levels of each influencing factor. Through this approach, the relative importance of ore-pass drift spacing, drawpoint spacing, the elevation difference between the extraction level and the undercut level on the stability of the roadway bottom structure can be clearly identified, thereby providing a reliable basis for the subsequent selection of key bottom-structure parameters. Table 10 presents the numerical simulation results for Schemes 1–9. The roadway deformation and plastic zone volume listed in the table were analyzed separately using the range analysis method.

**Table 10:** Roadway surrounding rock deformation and plastic zone volume of the bottom structure after the formation of the ore pass for each scheme.

Number	Ore-pass drift spacing A	Drawpoint spacing B	Elevation difference between the extraction level and the undercut level C	Roadway deformation (mm)	Plastic zone volume (m^3^)
1	28 (1)	16 (1)	18 (1)	294.14	302,180
2	28 (1)	18 (2)	20 (2)	245.36	262,015
3	28 (1)	20 (3)	22 (3)	218.96	224,932
4	32 (2)	16 (1)	22 (3)	210.04	190,844
5	32 (2)	18 (2)	20 (2)	223.27	205,235
6	32 (2)	20 (3)	18 (1)	239.08	227,566
7	36 (3)	16 (1)	20 (2)	227.13	228,572
8	36 (3)	18 (2)	22 (3)	209.06	201,301
9	36 (3)	20 (3)	18 (1)	243.15	247,863

Table 11 presents the range analysis results of roadway surrounding rock deformation. It can be observed from Table 11 that the influence of each factor on roadway deformation follows the order C > A > B. Among them, the elevation difference between the ore-drawing level and the undercutting level C exhibits the largest range value (*R* = 46.09), indicating that this parameter has the most significant effect on controlling roadway deformation. The ore-drawing crosscut spacing A has an R value of 28.68, ranking second in influence, while the ore-drawing drift spacing B shows the smallest R value of 17.86, indicating a relatively weak effect on roadway deformation. Based on the K_avg_ values at different levels of each factor, the optimal levels for factors A, B, and C are A₂, B₂, and C₃, respectively. Therefore, the optimal parameter combination based on the roadway deformation index is A₂B₂C₃.

**Table 11:** Range analysis results of surrounding rock deformation of the ore-pass drift roadway.

Item	Level	Ore-pass drift spacing A	Drawpoint spacing B	Elevation difference between the extraction level and the undercut level C
K value	1	758.43	731.28	776.34
	2	672.39	677.69	695.76
	3	679.34	701.19	638.06
K_avg_ vlaue	1	252.81	243.76	258.78
	2	224.13	225.9	231.92
	3	226.45	233.73	212.69
Optimal level		2 (32)	2 (18)	3 (22)
R		28.68	17.86	46.09
Number of levels		3		
Number of repetitions at each level r		3		

The range analysis results of the plastic zone volume of the bottom structure are presented in Table 12. It can be observed that the influence of each factor on the plastic zone volume follows the order A > C> B. Among them, the ore-drawing crosscut spacing A exhibits the largest range value (*R* = 5.52 × 10⁴ m³), indicating that it has the most significant effect on the plastic zone volume of the bottom structure. The elevation difference between the ore-drawing level and the undercutting level C shows a similar influence, with an R value of 5.35 × 10⁴ m³. In contrast, the ore-drawing drift spacing B has the smallest range value (*R* = 1.77 × 10⁴ m³), indicating a relatively weaker influence on the plastic zone volume. Based on the K_avg_ values at different levels of each factor, the optimal levels of A, B, and C are all A₂, B₂, and C₃, respectively. Therefore, the optimal parameter combination based on the plastic zone volume criterion is also A₂B₂C₃.

**Table 12:** Range analysis results of the plastic zone volume of the bottom structure.

Item	Level	Ore-pass drift spacing A	Drawpoint spacing B	Elevation difference between the extraction level and the undercut level C
K value	1	789,127	721,596	777,609
	2	623,645	668,551	695,822
	3	677,736	700,361	617,077
K_avg_ value (1.0e + 05)	1	2.6304	2.4053	2.592
	2	2.0788	2.2285	2.3194
	3	2.2591	2.3345	2.0569
Optimal level		2 (32)	2 (18)	3 (22)
R (1.0e + 04)		5.5161	1.7682	5.3511
Number of levels		3		
Number of repetitions at each level r		3		

Considering both evaluation indices roadway surrounding rock deformation and plastic zone volume of the bottom structure in Tables 11 and 12, it can be observed that although the sensitivity rankings of factors A and C differ slightly between the two indices, the optimal parameter levels identified are fully consistent. Therefore, the optimal parameter combination for this study is determined as A₂B₂C₃, corresponding to an ore-drawing crosscut spacing of 32 m, an ore-drawing drift spacing of 18 m, and an elevation difference of 22 m between the ore-drawing level and the undercutting level.

## Conclusions

Through a combined approach of engineering analogy, theoretical analysis, and numerical simulation, this study investigated the bottom structure layout and key parameters for block caving under ultra-deep and high-stress conditions. The main conclusions are as follows:

1. The branched herringbone layout demonstrates clear advantages over traditional square and Teniente-type layouts. It improves geometric coverage, reduces ore loss between draw zones, and better matches the turning characteristics of LHD equipment. Mechanistically, it optimizes gravity flow paths by reducing drift intersection angles, achieving a balance between ore recovery efficiency and equipment mobility.
2. Numerical simulations reveal that surrounding rock response evolves through three stages: initial stress concentration during draw drift excavation, rapid tensile damage after undercutting, and localized shear failure during ore-pass formation. Based on multi-index evaluation, the optimal parameters are: drawpoint drift spacing of 32 m, draw drift spacing of 18 m, and a 20 m vertical distance. This configuration forms a stable large peach-shaped pillar, ensuring effective load-bearing and roadway stability.
3. A dynamic stress control strategy combining advancing undercutting and pre-slotting is proposed. Maintaining an advance distance of 30–36 m creates a stress-relief zone, significantly reducing construction risk. An undercut step of 6.0 m achieves a balance between blasting efficiency and controlled stress release, effectively addressing the conflict between high in-situ stress and excavation safety.
4. Compared with Teniente and Henderson layouts, the proposed scheme shows comprehensive advantages: ore extraction efficiency is improved (transport distance reduced by approximately 15%), structural stability is enhanced (plastic zone reduced by approximately 20%), and construction cost is lowered by 10%-15%. This demonstrates that the proposed layout provides an effective solution for improving efficiency, safety, and economy in ultra-deep block caving operations.

Due to limitations in computational efficiency and the availability of experimental data, the rock mass in this study is assumed to be isotropic and modeled using the Mohr–Coulomb constitutive model, without considering the effects of parameter variability and strain-softening; in future work, more comprehensive rock mass parameters will be incorporated to develop discrete element models accounting for anisotropy and jointed structures, and the results will be compared with field observations to better support practical mining operations.

## Data Availability

The datasets used and analysed during the current study available from the corresponding author on reasonable request.
